# Tunnel vs. coronally advanced flap in combination with a connective tissue graft for the treatment of multiple gingival recessions: a multi-center randomized clinical trial

**DOI:** 10.1007/s00784-023-04975-7

**Published:** 2023-03-29

**Authors:** Jerián González-Febles, Mario Romandini, Florencia Laciar-Oudshoorn, Fernando Noguerol, Crystal Marruganti, Antonio Bujaldón-Daza, Ion Zabalegui, Mariano Sanz

**Affiliations:** 1 Section of Post-Graduate Periodontology, Faculty of Odontology, Department of Dental Clinical Specialties, University Complutense, Plaza Ramón Y Cajal, 3, 28040 Madrid, Spain; 2 Clínica Periodontal Antonio Bujaldón, Almería, Spain; 3 Unit of Periodontology, Endodontology and Restorative Dentistry, Department of Medical Biotechnologies, University of Siena, Siena, Italy

**Keywords:** Gingival recession, Clinical trial, Dentistry, Minimally invasive surgical procedures, Mucogingival surgery, Root coverage procedures, Patient-reported outcome measures

## Abstract

**Objective:**

To evaluate the efficacy of the partial-thickness non-advanced tunnel technique (TUN) versus the coronally advanced flap (CAF), both combined with a connective tissue graft, in the treatment of multiple gingival recessions.

**Materials and methods:**

Twenty-nine patients (83 teeth) affected by multiple gingival recessions were treated in two clinical centers with either the test (TUN) or the control (CAF) intervention combined with a connective tissue graft. Outcomes at 3 and 6 months after surgery included complete root coverage (CRC—primary outcome), mean root coverage (mRC), changes in recession depth (RD), probing pocket depth (PPD), and keratinized tissue height (KT). Root sensitivity and root coverage esthetic score (RES) were also evaluated at 6-month examination. Surgery duration, wound healing index (WHI), and patient-reported outcome measures (PROMs) were additionally considered.

**Results:**

At 6 months, CRC was observed in 80.9% and 79.5% of the teeth treated with TUN and CAF, respectively (odds ratio = 1.2; *p* = 0.802). No differences between groups were also observed in terms of mRC (TUN = 94.0%; CAF = 91.1%), RD and PPD reductions, root sensitivity, RES, and WHI. KT increase was significantly higher in teeth treated with TUN (Difference in Means – MD =  − 1.0 mm; *p* = 0.001). Surgery duration was shorter (MD =  − 19.3 min; *p* = 0.001), and patients reported less intra-surgical pain (MD =  − 16.4; *p* = 0.028) as well as postoperative morbidity in TUN compared with CAF.

**Conclusions:**

Both surgical interventions showed a similar efficacy in terms of root coverage, albeit TUN was associated with a higher increase in KT and with a milder patient’s surgical experience.

**Clinical relevance:**

Both techniques have shown similar efficacy for the coverage of exposed root surfaces, although clinicians may consider TUN as less invasive.

**Trial registration:**

Clinicaltrials.gov (NCT05122468)

**Supplementary Information:**

The online version contains supplementary material available at 10.1007/s00784-023-04975-7.

## Introduction

Gingival recession (GR) is a clinical condition defined by the apical shift of the gingival margin below the cemento-enamel junction (CEJ). It is associated with attachment loss and with exposure of the root surface. When the loss of attachment and marginal tissue recession are predominantly found at buccal surfaces, these lesions are usually present in populations with high standards of oral hygiene [1, 2], and, if untreated, they tend to progress [3]. The recent 2017 World Workshop on the Classification of Periodontal and Peri-implant Diseases has adopted a classification of gingival recessions [4] based on the dimensions of the buccal/lingual gingival recession in relation to the interdental clinical attachment loss [5]. Although the prevalence of GR in adults is high (around 90%) [6], only 12% of the population is affected by localized gingival recessions (LGR) with intact interdental tissues (RT1) GR and only 3.1% by multiple adjacent RT1 GR [2, 7]. LGR-affected patients usually complain of esthetic concerns, although the root surface exposure may also lead to secondary hypersensitivity, caries, and discomfort [8].

The treatment of LGR aims to reconstruct the lost soft tissues and to fully cover the exposed roots. With this purpose, several surgical techniques have been proposed combining different surgical designs (flaps and tunneling approaches) and grafting materials (autologous, allogenic, or xenogeneic) [9–15]. Recent consensus workshops have identified the coronally advanced flap (CAF) (Zucchelli & Sanctis, 2000) as the gold standard technique for the treatment of multiple adjacent LGRs [9, 10]. This surgical technique can be used alone, although better long-term results have been reported when it is combined with a CTG [12, 16–18].

Tunneling techniques (TUN), always in combination with a CTG, have also demonstrated high clinical efficacy in the treatment of multiple adjacent LGRs [12, 19–24]. This surgical approach, however, has been modified by different authors depending on the type of the submarginal dissection (split versus full thickness) and the gingival margin position with respect to the CTG (non-advanced exposing the coronal part of the CTG or coronally/laterally moved to fully cover the CTG) [21, 23, 25, 26].

The existing randomized clinical trials (RCTs) comparing CAF with TUN for the treatment of multiple adjacent LGRs have reported conflicting results, probably due to patient and site selection (combination of single and multiple LGRs) [27–29] and to the use of different TUN approaches [28, 30]. It was, therefore, the purpose of this 6-month dual-center RCT to evaluate the efficacy of a specific TUN technique (the partial-thickness non-advanced [21] versus the CAF, both combined with a CTG, for the treatment of multiple LGRs.

## Material and methods

This manuscript is reported following the Consolidated Standards of Reporting Trials (CONSORT) 2010 guidelines [31]. The protocol of the study was registered in Clinicaltrials.gov (NCT05122468) and approved by the Ethical Committee of Hospital Clínico San Carlos of Madrid (Internal code: 16/504). All participants were informed in detail on the objectives of this investigation and on the surgical procedures utilized. Patients entered in the study after signing an informed consent and agreeing to comply with the study protocol.

### Trial design

This study was designed as a randomized dual-center clinical trial with two parallel groups (1:1 allocation ratio) and a 6-month follow-up.

### Participants

Study subjects were consecutively recruited from patients attending two specialized periodontal clinics: (1) the Post-Graduate Periodontology Clinic at the Faculty of Odontology, Complutense University (Madrid, Spain) and (2) the specialist Periodontal Clinic—Antonio Bujaldón (Almería, Spain) between October 2016 and May 2021.

Adult subjects (≥ 18 years) were included if presenting a minimum of 2 and a maximum of 4 adjacent RT1/RT2 GRs, at least one of them with a depth > 2 mm. RT2 GRs were only considered eligible when the interdental CEJ was not exposed. Subjects were not eligible if they:

• suffered from systemic diseases contraindicating elective surgery
• were affected by untreated periodontitis
• were current smokers (self-reported)
• presented inadequate level of self-performed oral hygiene (full-mouth plaque or bleeding scores ≥25%)

Local exclusion criteria were furcation involvement, uncorrected trauma from toothbrushing, severe tooth malposition, and history of root coverage procedures.

### Surgical interventions

All surgical interventions were carried out by two trained specialists (MR and JGF). Prior to the intervention, the CEJ was identified in the selected teeth; if not readily present, it was reconstructed with adhesive dental materials using the adjacent and contralateral teeth as reference. Conversely, any restorative materials invading the root surface was removed up to the CEJ.

**Fig. 1:**
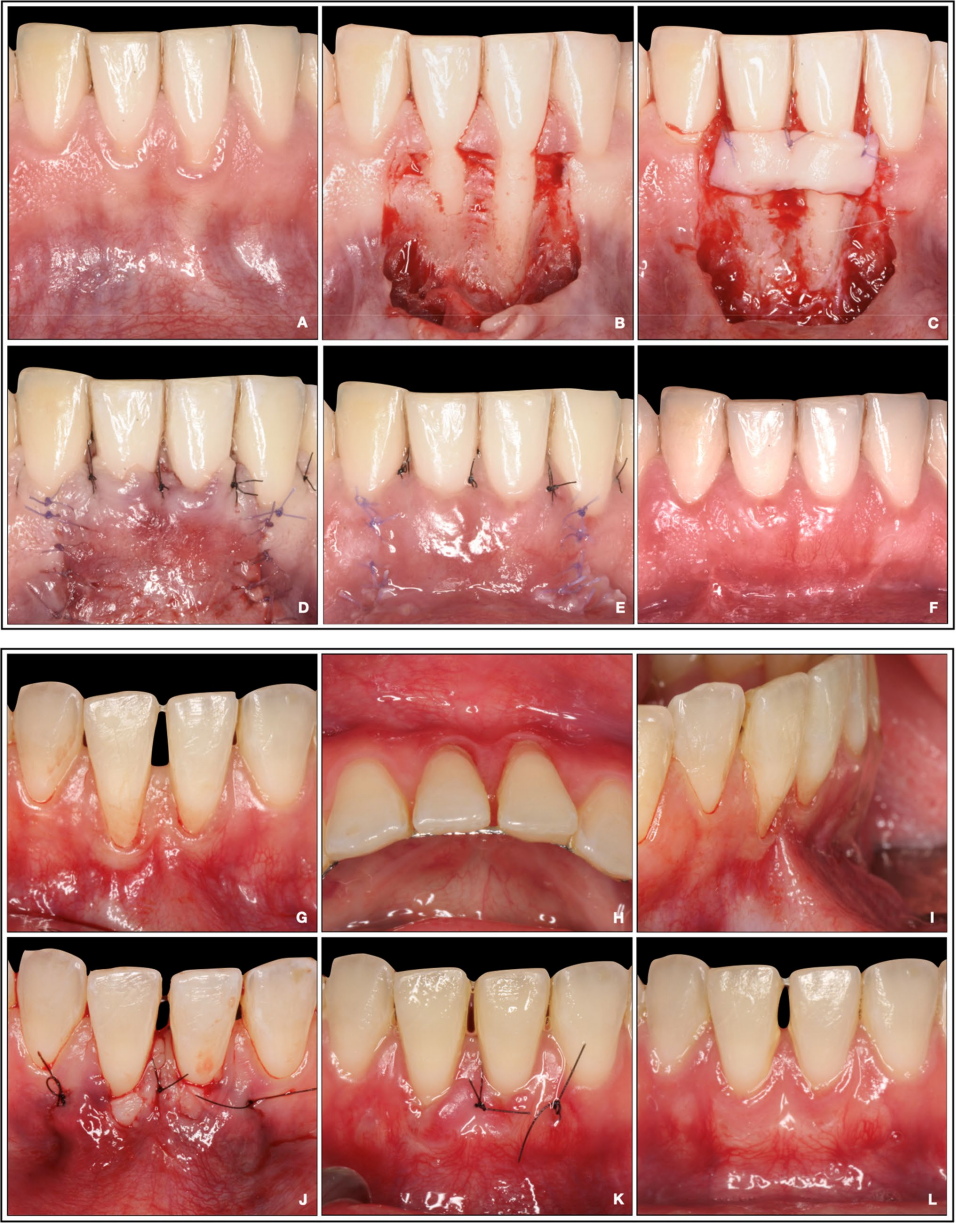
Images illustrating interventions by group allocation. **A**–**F** Study case treated with a CAF: **A** pre-operative view; **B** flap elevation; **C** CTG sutured; **D** flap sutured; **E** 10 days; **F** 6 months. **G**–**L** Study case treated with TUN: **G**, **H**, **I** pre-operative view; **J** CTG sutured; **K** 10 days; **L** 6 months. CAF, coronally advanced flap; CTG, connective tissue graft; TUN, split-thickness non-advanced tunnel

The interventions started in all included subjects with a CTG harvesting from the palatal mucosa through the de-epithelized free gingival graft (FGG) technique [32]. In brief, a FGG of 1–1.5 mm in thickness and extending at least 3 mm from the gingival margins of the affected teeth in length/height was harvested from the lateral posterior palatal mucosa. Once the donor area was protected with a collagen sponge and sutured, the FGG graft was carefully de-epithelized and placed in saline solution. Then, the subjects were randomized using a block randomization list (in blocks of 4 participants, stratified by study center) to one of the following groups (Fig. 1):

- TUN + CTG (test group)

- CAF + CTG (control group)

#### *Test intervention: TUN* + *CTG* [21]

With the use of a micro-surgical blade (Mini-Crescent® 1.25-mm micro-surgical blade, Sharpoint), a single-plane split-thickness dissection was done, extending at least 5 mm in all directions from the gingival margins. In the interdental spaces, the undersurface of the buccal interdental papillae was dissected extending coronally up to 2 mm from the CEJ. After instrumenting the exposed tooth surfaces with curettes, the CTG was positioned inside the tunnel and secured in place with two internal vertical mattress sutures at both mesial and distal ends, locating its coronal end 1 mm above the CEJ. No attempts were made to advance the marginal tissues: consequently, the most coronal portion of the CTG was intentionally left exposed. However, when large amounts of the CTG resulted exposed, the mesial and distal gingival marginal ends of the involved recessions were approximated through interrupted sutures. 

#### *Control intervention: CAF* + *CTG* [33–35]

A split-full-split flap was elevated to allow its passive coronal advancement. Whenever possible, vertical releasing incisions were avoided. Once the exposed root surfaces were instrumented with curettes, the anatomical interdental papillae were de-epithelialized, and the CTG was sutured to these papillae aiming to secure the coronal end of the graft at the level of the CEJ, using single interrupted resorbable sutures (6/0 to 8/0). Finally, the flap was advanced and sutured 1 mm coronally to the CEJ through sling sutures, closely adapting every surgical papilla to the correspondent de-epithelized anatomic papilla and entirely covering the CTG.

### Post-surgical care

Patients were given anti-inflammatory and pain medication (Ibuprofen 600 mg) and were advised to take them as required, up to a maximum of 3 tablets/day. When contraindicated, paracetamol (500 mg) was used instead. Patients were also instructed to avoid any self-performed mechanical oral hygiene procedure or trauma on the surgical area for 14 days, and during 4 weeks, they were instructed to rinse with 0.12% chlorhexidine digluconate + 0.05% cetylpyridinium chloride for 1 min twice a day. Sutures were removed at 7–10 days after surgery. Thereafter, during the first month, the patients were recalled weekly for oral hygiene reinforcement and professional supra-gingival plaque removal and then at the 3- and 6-month follow-up visits.

### Outcomes

The primary outcome of this RCT was the percentage of complete root coverage (CRC) at 6 months. As secondary endpoints, the mean percentage (%) of root coverage (mRC) and the changes between baseline and 3/6 months in recession depth (both GM-CEJ and IE-GM), probing pocket depth (PPD), and width of keratinized tissue (KT) were measured. Furthermore, root sensitivity, duration of the surgical intervention, early wound healing, root coverage esthetic score (RES), and patient-reported outcomes (PROMS) were assessed. Except for the PROMs, the remaining outcomes were assessed by two examiners (MR and JG-F).

#### Clinical variables

At baseline and 3/6 months postoperatively, the following clinical parameters were measured at the buccal aspect of each included tooth at the site of the deepest recession, using a UNC15 periodontal probe: distance from the gingival margin to both the cemento–enamel junction (GM-CEJ) and the incisal/occlusal edge (IE-GM), probing pocket depth (PPD), and width of KT. In addition, at baseline and 6 months postoperatively, full-mouth plaque and bleeding scores (FMPS/FMPS) were measured at 4 sites per tooth. At the same time-points, root sensitivity was evaluated after a blow of air with the unit syringe (air test) and after applying tactile contact with the tip of the periodontal probe (probe test) [36]. The surgical intervention duration after randomization was also measured and noted.

#### Early wound healing

Early wound healing was assessed at 1-, 2-, and 4-week follow-ups evaluating dichotomously the CEJ coverage and the presence of edema/inflammation, fibrin accumulation, marginal necrosis, and obvious mobility of the wound margin on the root surface. A wound healing score was then generated by adding these five items [37, 38].

#### Root coverage esthetic score (RES)

At the 6-month examination, the esthetic outcomes were assessed using the “root coverage esthetic score” (RES) [39]. Briefly, the following five parameters were evaluated: gingival margin level (0 = failure of root coverage; 3 = partial root coverage; 6 = complete root coverage), marginal tissue contour (0 = irregular; 1 = proper), soft tissue texture (0 = presence of scar/keloid; 1 = absence of scar/keloid), mucogingival junction (MGJ) (0 = MGJ not aligned; 1 = MGJ aligned), and gingival color (0 = color differs from adjacent teeth; 1 = normal color and integration). A total RES score was calculated by adding these 5 components.

#### Patient-reported outcome measures (PROMs)

Self-reported patient perception and pain during the surgical intervention were evaluated immediately after surgery using a 100-mm visual analogue scale (VAS), being its extremes “no pain” and “most severe pain possible.” Afterwards the patients were instructed to use a postoperative diary during the first 14 days to collect (a) postoperative pain by filling the same VAS scale, (b) the number of tablets used of the provided anti-inflammatory and pain medication, (c) an oral-health quality of life questionnaire (OHIP-14 – [40], and (d) another open-ended questionnaire referring the patient-reported experiences (PREMs) including post-surgery sequelae, pain and discomfort, oral function, and interference with daily activities [41]. At 6 months, patients were asked to report (a) their overall oral-related quality of life by filling again the OHIP-14 questionnaire and (b) their condition-specific health-related quality of life by filling a specific HRQoL questionnaire consisting on a 5-point Likert scale assessing the patient’s level of concern in terms of esthetics, sensitivity to cold, sensitivity to brushing, tooth wear, dental caries, and fear to lose the involved teeth [38]. Overall OHIP-14 and HRQoL scores were also calculated by adding their respective components.

### Data analysis

A sample size of 30 patients was targeted for detecting a difference of 35% in CRC (number needed to treat = 3) at 6 months between the test and control group, using a multilevel logistic regression analysis adjusted for clustering (with an expected mean cluster size of 2.5 teeth/patient), with an 80% power and a critical level of significance of 0.05.

Descriptive characteristics of the study participants and teeth were expressed for continuous variables as mean (standard deviation—SD), while for categorical variables as number (percentage—%). Results were expressed as differences in means (MD) or odds ratios (OR), with 95% confidence intervals (CI). Differences between groups for tooth-level variables were tested through multilevel logistic (binary) or linear (continuous) regression analyses adjusted for clustering. Patient-level variables were compared between groups applying the χ2 test for binary variables and unpaired Student’s t-test for continuous ones. All comparisons between groups were carried out using 2-sided hypothesis and an alpha < 0.05 level of significance.

Data analysis was performed by a blinded statistician using STATA version 17.1 software (StataCorp LLC, Texas, USA) and applying the intention-to-treat principle.

## Results

Thirty patients (87 teeth) were randomized, 15 to the test and 15 to the control group (42 and 45 teeth, respectively) (Figure S1). This sample consisted mainly of female patients (73.3%) and had a mean age of 34.3 (± 9.4) years, being most of the included teeth incisors (41.4%). The mean baseline recession depth was 2.8 (± 1.5) mm, and in 80.0% of the teeth hypersensitivity was positive to the air test (Table 1). One patient (4 teeth) from the control group did not receive the intervention, due to withdrawal of the consent to participate in the study.

**Table 1:** General characteristics of the study patients (*N* = 30) and teeth (*N* = 87)

*Patient-level characteristics*	Overall (*N* = 30)	CAF (*N* = 15)	TUN (*N* = 15)
**Age** (years), mean (± SD)	34.3 (± 9.4)	34.7 (± 9.5)	34.0 (± 9.5)
Gender, *N* (%)			
* Males*	8 (26.7)	5 (33.3)	3 (20.0)
* Females*	22 (73.3)	10 (66.7)	12 (80.0)
**FMPS**, mean (± SD)	6.6 (± 6.2)	7.3 (± 6.6)	5.9 (± 5.8)
**FMBS**, mean (± SD)	7.7 (± 8.8)	7.9 (± 10.5)	7.5 (± 7.3)
**Root sensitivity-air test positive**, *N* (%)	24 (80.0)	12 (80.0)	12 (80.0)
**Root sensitivity-probe test positive**, *N* (%)	14 (46.7)	6 (40.0)	8 (53.3)
**OHIP-14**, mean (± SD)	6.5 (± 3.7)	6.7 (± 3.8)	6.3 (± 3.8)
**Condition-specific HRQoL**, mean (± SD)	19.2 (± 3.8)	18.7 (± 3.8)	19.7 (± 2.8)
***Tooth-level characteristics***	**Overall (*N* = 87)**	**CAF (*N* = 45)**	**TUN (*N* = 42)**
**Tooth position**, *N* (%)			
* Incisor*	36 (41.4)	11 (12.6)	25 (28.7)
* Canine*	17 (19.5)	10 (11.5)	7 (8.0)
* Premolar*	28 (32.2)	18 (20.7)	10 (11.5)
* Molar*	6 (6.9)	4 (4.6)	2 (2.3)
**Arch**, *N* (%)			
* Maxilla*	36 (41.3)	34 (39.1)	2 (2.3)
* Mandible*	51 (58.6)	11 (12.7)	40 (45.9)
**Side**, *N* (%)			
* Right*	33 (37.9)	11 (12.6)	22 (25.3)
* Left*	54 (62.1)	34 (39.1)	20 (22.9)
**Recession depth (GM-CEJ)** (mm), mean (± SD)	2.8 (± 1.5)	2.8 (± 1.1)	2.8 (± 1.8)
**Recession depth (IE-GM)** (mm), mean (± SD)	11.8 (± 1.8)	11.7 (± 1.7)	11.8 (± 2.09)
**PPD** (mm), mean (± SD)	1.7 (± 0.6)	1.7 (± 0.6)	1.8 (± 0.6)
**KT** (mm), mean (± SD)	2.5 (± 1.3)	2.6 (± 1.2)	2.3 (± 1.3)

*CAF* coronally advanced flap, *CEJ* cemento enamel junction, *IE* incisal edge, *FMBS* full mouth bleeding score, *FMPS* full mouth plaque score, *GM* gingival margin, *KT* keratinized tissue, *mm* millimeter, *N* number, *PPD* probing pocket depth, *TUN* partial-thickness/non-advanced tunnel, *SD* standard deviation; %, percentage

### Clinical outcomes

Table 2 depicts the clinical outcomes at tooth level, while Table 3 presents the patient-level results. At 6 months, CRC was achieved in 80.9% and 79.5% of the treated teeth treated with TUN and CAF, respectively (OR = 1.2; 95% CI: 0.3/5.8; *p* = 0.802). The mean recession coverage was also similar between groups (TUN = 94.0 ± 14.1%; CAF = 91.1 ± 18.8%; *p* = 0.547). Likewise, the mean recession depth (GM-CEJ) reduction was 2.7 ± 1.8 mm and 2.5 (± 1.1) in the test and control groups, respectively (MD = 0.2; 95% CI: − 0.5/1.1; *p* = 0.462). The increase in KT was significantly higher in TUN (1.4 ± 1.4 mm) compared to CAF (0.4 ± 0.7 mm) (MD =  − 1.0 mm; 95%: − 1.8/ − 0.4; *p* = 0.001).

**Table 2:** Clinical outcomes of the included recessions (tooth level)

	**Overall** (*N* = 83)	**CAF** (*N* = 42)	**TUN** (*N* = 41)	**Effect size**	***p*-value**
**Recession depth (GM-CEJ) change** (mm), mean (± SD)					
Baseline—3 m	2.6 (± 1.4)	2.5 (± 1.0)	2.7 (± 1.8)	MD = 0.2 (95% CI: − 0.6/0.9)	0.612
Baseline—6 m	2.6 (± 1.5)	2.5 (± 1.1)	2.7 (± 1.8)	MD = 0.2 (95% CI: − 0.5/1.1)	0.462
**Recession depth (IE-CEJ)** change (mm), mean (± SD)					
Baseline—3 m	2.6 (± 1.2)	2.7 (± 1.0)	2.5 (± 1.4)	MD = − 0.1 (95% CI: − 0.7/0.4)	0.626
Baseline—6 m	2.6 (± 1.3)	2.7 (± 1.0)	2.6 (± 1.5)	MD = − 0.05 (95% CI: − 0.7/0.6)	0.869
**PPD change** (mm), mean (± SD)					
Baseline—3 m	− 0.1 (± 0.5)	− 0.1 (± 0.3)	− 0.01 (± 0.7)	MD = 0.1 (95% CI: − 0.2/0.4)	0.628
Baseline—6 m	− 0.01 (± 0.6)	− 0.1 (± 0.5)	0.05 (± 0.6)	MD = 0.1 (95% CI: − 0.1/0.3)	0.367
**KT change** (mm), mean (± SD)					
Baseline—3 m	− 0.9 (± 1.3)	− 0.3 (± 0.9)	− 1.5 (± 1.4)	MD = − 1.2 (95% CI: − 1.9/ − 0.5)	0.001*
Baseline—6 m	− 0.9 (± 1.2)	− 0.4 (± 0.7)	− 1.4 (± 1.4)	MD = − 1.0 (95% CI: − 1.8/ − 0.4)	0.001*
**mRC** (%), mean (± SD)					
Baseline—3 m	93.2 (± 16.3)	93.5 (± 16.4)	92.9 (± 16.5)	MD = − 1.4 (95% CI: − 11.2/8.2)	0.767
Baseline—6 m	92.6 (± 16.5)	91.1 (± 18.8)	94.0 (± 14.1)	MD = 2.7 (95% CI: − 6.2/11.7)	0.547
**CRC**, *N* (%)					
3 m	67 (80.7)	34 (82.9)	33 (78.6)	OR = 0.6 (95% CI: 0.1/3.5)	0.583
6 m	66 (79.5)	32 (78.1)	34 (80.9)	OR = 1.2 (95% CI: 0.3/5.8)	0.802

*CAF* coronally advanced flap, *CEJ* cemento enamel junction, *CI* confidence interval, *CRC* complete root coverage, *IE* incisal edge, *GM* gingival margin, *KT* keratinized tissue, *m* months, *mm* millimeter, *MD* mean difference, *mRC* mean root coverage, *OR* odds ratio, *N* number, *PPD* probing pocket depth, *TUN* partial-thickness/non-advanced tunnel, *SD* standard deviation; %, percentage

**Table 3:** Clinical outcomes of the included patients (patient level)

	**Overall** (*N* = 29)	**CAF** (*N* = 14)	**TUN** (*N* = 15)	**Effect size**	***p*-value**
**FMPS change** (%), mean (± SD)					
Baseline—3 m	− 6.9 (± 10.9)	− 7.5 (± 12.5)	− 6.5 (± 9.8)	MD = 0.9 (95% CI: − 7.7/9.6)	0.824
Baseline—6 m	− 5.5 (± 10.1)	− 4.2 (± 5.9)	− 6.6 (± 12.8)	MD = − 2.5 (95% CI: − 10.4/5.5)	0.530
**FMBS change** (%), mean (± SD)					
Baseline—3 m	− 4.8 (± 8.8)	− 7.3 (± 10.9)	− 2.6 (± 5.9)	MD = 4.8 (95% CI: − 1.9/11.5)	0.156
Baseline—6 m	− 5.5 (± 11.5)	− 6.3 (± 12.1)	− 4.8 (± 11.4)	MD = 1.6 (95% CI: − 7.5/10.7)	0.726
**Root sensitivity-air test positive** *(6-months)*, *N* (%)	4 (13.8)	2 (14.3)	2 (13.3)	OR = 0.9 (95% CI: 0.1/7.6)	0.941
**Root sensitivity-probe test positive** *(6-months)*, *N* (%)	1 (3.5)	1 (7.1)	0 (0.0)	NE	NE
**Surgery duration** (min), mean (± SD)	53.9 ± 16.3	63.9 ± 18.0	44.5 ± 6.4	MD = − 19.3 (95% CI: − 29.5/ − 9.2)	0.001*

CAF coronally advanced flap, *CI* confidence interval, *FMBS* full mouth bleeding score, *FMPS* full mouth plaque score, *m* months, *MD* mean difference, *min* minutes, *N* number, *NE* not estimable, *TUN* partial-thickness/non-advanced tunnel, *SD* standard deviation

^*^Statistically significant

While root sensitivity disappeared in most of the patients, 2 patients in the test and 2 in the control groups were still positive to air test at the 6-month examination (OR = 0.9; 95% CI: 0.1/7.6; *p* = 0.941). Surgery duration was significantly shorter in TUN compared to CAF (MD =  − 19.3 min; 95% CI: − 29.5/ − 9.2; *p* = 0.001).

### Early wound healing

Table 4 reports the results on early wound healing at 1, 2, and 4 weeks after surgery. A higher percentage of sites with edema/inflammation and marginal mobility was observed in CAF than in TUN, but differences were not statistically significant. During early healing (1–4 weeks), CEJ was uncovered significantly more frequently in TUN versus CAF (4 weeks: OR = 17.2; 95%: 1.3–229.4; *p* = 0.032). The total wound healing index showed no significant differences between groups (MD = 0.1; 95% CI: − 0.3/0.6; *p* = 0.562).

**Table 4:** Wound healing at 1, 2, and 4 weeks postoperatively (tooth level)

	**Overall** (*N* = 83)	**CAF** (*N* = 42)	**TUN** (*N* = 41)	**Effect size**	***p*-value**
**CEJ uncovered**, *N* (%)					
1 week	16 (19.3)	1 (2.4)	15 (35.7)	OR = 31.2 (95% CI: 2.6/376.8)	0.007*
2 weeks	16 (19.3)	3 (7.3)	13 (30.9)	OR = 8.2 (95% CI: 1.1/63.6)	0.041*
4 weeks	22 (26.5)	4 (9.8)	18 (42.9)	OR = 17.2 (95% CI: 1.3/229.4)	0.032*
**Edema/inflammation**, *N* (%)					
1 week	53 (63.9)	31 (75.6)	22 (52.4)	OR = 0.2 (95% CI: 0.01/2.6)	0.197
2 weeks	32 (38.6)	26 (63.4)	6 (14.3)	OR = 0.001 (95% CI: 0.0001/3.8)	0.107
4 weeks	9 (10.8)	6 (14.6)	3 (7.1)	OR = 0.4 (95% CI: 0.03/43.7)	0.693
**Fibrin accumulation,** *N* (%)					
1 week	26 (31.3)	11 (26.8)	15 (35.7)	OR = 2.1 (95% CI: 0.3/13.2)	0.433
2 weeks	7 (8.4)	4 (9.8)	3 (7.1)	OR = 1.1 (95% CI: 0.03/35.8)	0.957
4 weeks	6 (7.2)	3 (7.3)	3 (7.1)	OR = 0.61 (95% CI: 0.01/227.8)	0.870
**Marginal necrosis,** *N* (%)					
1 week	8 (9.6)	4 (9.8)	4 (9.5)	OR = 0.9 (95% CI: 0.2/4.6)	0.945
2 weeks	3 (3.6)	1 (2.4)	2 (4.8)	OR = 1.6 (95% CI: 0.04/72.4)	0.805
4 weeks	0 (0.0)	0 (0.0)	0 (0.0)	NE	NE
**Marginal mobility,** *N* (%)					
1 week	14 (16.9)	9 (21.9)	5 (11.9)	OR = 0.3 (95% CI: 0.02/5.0)	0.434
2 weeks	15 (18.1)	9 (21.9)	6 (14.3)	OR = 0.2 (95% CI: 0.01/4.9)	0.351
4 weeks	7 (8.4)	5 (12.2)	2 (4.8)	OR = 0.35 (95% CI: 0.1/2.1)	0.252
**Wound healing index** (sum), mean (± SD)					
1 week	1.4 (± 1.1)	1.4 (± 1.0)	1.5 (± 1.2)	MD = 0.1 (95% CI: − 0.4/0.6)	0.720
2 weeks	0.9 (± 0.8)	1.0 (± 0.7)	0.7 (± 0.9)	MD = − 0.3 (95% CI: − 0.8/0.1)	0.179
4 weeks	0.5 (± 0.8)	0.4 (± 0.8)	0.6 (± 0.8)	MD = 0.1 (95% CI: − 0.3/0.6)	0.562

*CAF* coronally advanced flap, *CI* confidence interval, *MD* mean difference, *MGJ* muco-gingival junction, *CEJ* cemento enamel junction, *OR* odds ratio, *N* number, *NE* not estimable, *SD* standard deviation, *TUN* tunnel; %, percentage

^*^Statistically significant

### Root coverage esthetic score (RES)

Table 5 depicts the RES results 6 months after surgery. Better scores in soft tissue texture were recorded in CAF, but none of the RES components showed statistically significant differences between groups. The total RES score was 8.8 (± 1.5) and 8.8 (± 1.8) in the test and in the control group, respectively (MD =  − 0.02; 95% CI: − 0.9/0.9; *p* = 0.962).

**Table 5:** Root coverage esthetic score (RES) at 6 months (tooth level)

	**Overall (*N* = 83)**	**CAF (*N* = 42)**	**TUN (*N* = 41)**	**Effect size**	***p*-value**
**Gingival margin level,** *N* (%)					
Failure of root coverage (0)	0 (0.0)	0 (0.0)	0 (0.0)	-	-
Partial root coverage (3)	17 (20.5)	9 (21.9)	8 (19.1)	*Ref*	*Ref*
Complete root coverage (6)	66 (79.5)	32 (78.1)	34 (80.9)	NE	NE
**Marginal tissue contour,** *N* (%)					
Irregular (0)	8 (9.6)	5 (12.2)	3 (7.1)	*Ref*	*Ref*
Proper (1)	75 (90.4)	36 (87.8)	39 (92.9)	OR = 1.8 (95% CI: 0.3/10.7)	0.525
**Soft tissue texture,** *N* (%)					
Scar/keloid (0)	24 (28.9)	10 (24.4)	14 (33.3)	*Ref*	*Ref*
Absence of scar/keloid (1)	59 (71.1)	31 (75.6)	28 (66.7)	OR = 0.2 (95% CI: 0.02/3.6)	0.301
**MGJ,** *N* (%)					
MGJ not aligned (0)	14 (16.9)	7 (17.1)	7 (16.7)	*Ref*	*Ref*
MGJ aligned (1)	69 (83.1)	34 (82.9)	35 (83.3)	OR = 1.4 (95% CI: 0.1/14.5)	0.803
**Gingival color,** *N* (%)					
Color differs from adjacent (0)	4 (4.8)	1 (2.4)	3 (7.1)	*Ref*	*Ref*
Normal color and integration (1)	79 (95.2)	40 (97.6)	39 (92.9)	OR = 0.8 (95% CI: 0.001/5.9)	0.964
**Total RES score** (sum), mean (± SD)	8.8 (± 1.6)	8.8 (± 1.8)	8.8 (± 1.5)	MD = − 0.02 (95% CI: − 0.9/0.9)	0.962

*CAF* coronally advanced flap, *CI* confidence interval, *MD* mean difference, *MGJ* muco-gingival junction, *OR* odds ratio, *N* number, *TUN* partial-thickness/non-advanced tunnel, *SD* standard deviation, %, percentage

### Patient-reported outcome measures (PROMs)

Patients treated with TUN reported significantly less pain than CAF during the surgical intervention (MD =  − 16.4; 95% CI: − 31.0/ − 1.9; *p* = 0.028). Although the overall surgical experience was also better in TUN than in CAF, this difference was not statistically significant (MD =  − 16.0; 95% CI: − 34.7/2.6; *p* = 0.089) (Table S1).

**Fig. 2:**
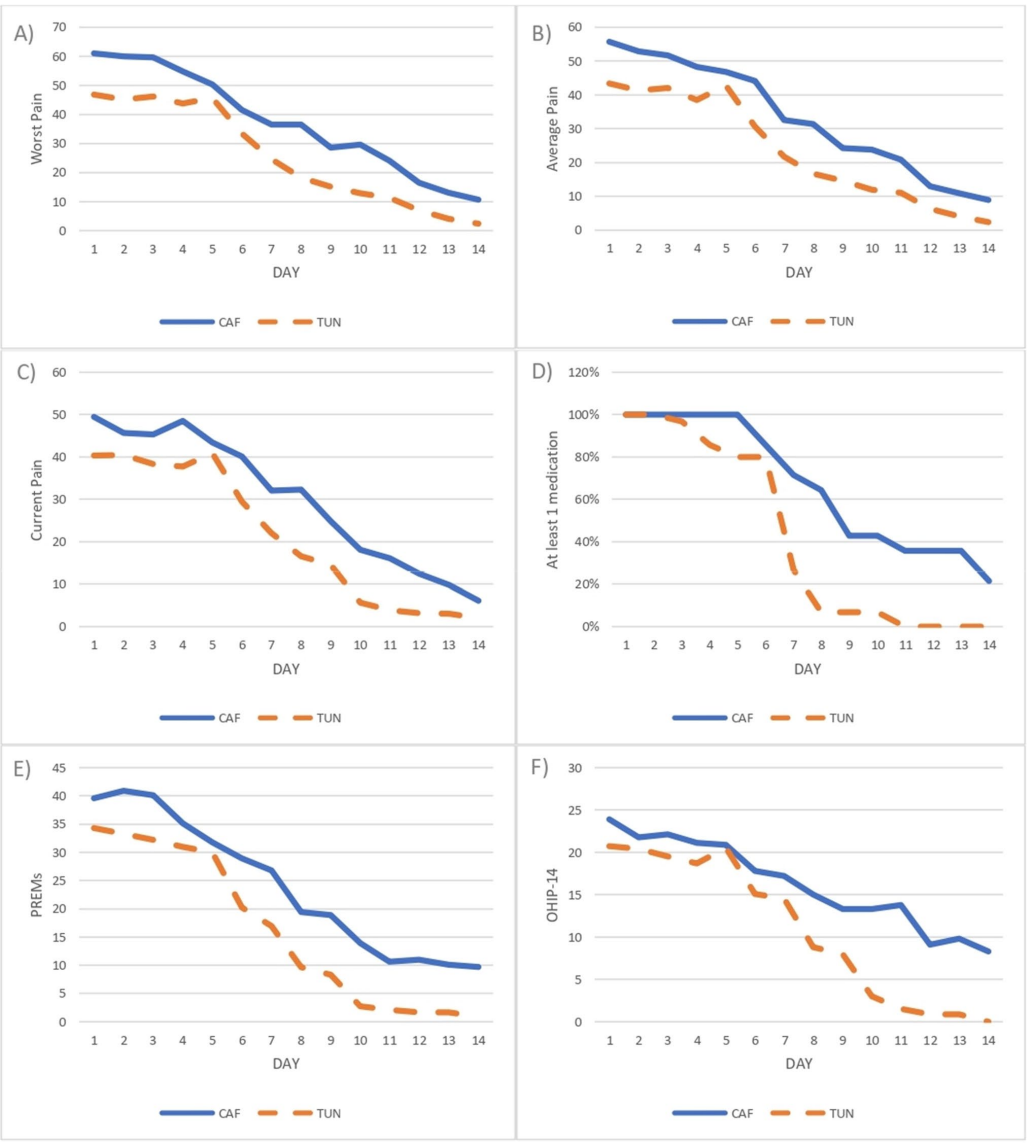
Self-reported pain, medications, PREMs, and OHIP-14 during the 14-day postoperative period. Blue continuous line: subjects treated with CAF. Orange dashed line: subjects treated with TUN. **A** Worst pain; **B** average pain; **C** current pain; **D** at least one medication (pain-killers and/or anti-inflammatory drugs); **E** PREMs; **F** OHIP-14. CAF, coronally advanced flap; OHIP-14, oral health-related quality of life-14; PREMs, patient-reported experience measures; TUN, split-thickness non-advanced tunnel; VAS, visual analogue scale

During the 2 weeks postoperative period, patients treated with TUN reported less pain compared with the ones treated with CAF (Fig. 2), but these differences were statistically significant only for “worst pain” at days 1, 10, and 11 (Tables S2, S3, S4). Patients treated with CAF also reported a significantly higher consumption of painkillers from week 1–2 compared with patients treated with TUN (Table S5). Consequently, TUN patients stopped painkillers consumption 3 days before than the CAF ones (MD =  − 3.1; 95% CI: − 5.4/ − 0.8; *p* = 0.011).

Patients treated with TUN reported also lower PREMs scores during the 2-week postoperative period, being the differences at days 10 and 14 statistically significant (Table S6). Similarly, TUN patients reported lower OHIP-14 values compared to the CAF ones between days 8–14, being these differences statistically significant at days 11, 13, and 14 (Table S7). At 6 months, OHIP-14 and condition-specific HRQoL did not differ between groups (Tables S8 and S9).

## Discussion

The results from the present study indicate that both surgical interventions (TUN and CAF) were of similar efficacy in terms of root coverage, early wound healing, and esthetic outcomes at 3 and 6 months. However, TUN demonstrated a significantly higher increase in KT and a shorter surgery duration. Similarly, PROMS were significantly better in patients treated with TUN during the first two postoperative weeks, although the overall and condition-specific quality of life resulted similar for both treatments at 6 months.

These results agree with three previous RCTs comparing TUN with CAF and reporting similar root coverage outcomes [28, 29]. However, in other RCTs, better root coverage results have been reported in either TUN [27] or CAF [30]. These discrepancies are possibly due to the inherent surgical and methodological study peculiarities, which resulted in low CRC rates with either CAF (21%—[27] or TUN (31%—[30]).

In the present RCT, the only significant difference in the clinical outcomes occurred for the increase in KT, which was in favor of TUN. This finding is also consistent with three previous reports [27–29], although in one other trial the results were opposite, with significantly higher KT increase in CAF compared to TUN [30]. This discrepancy may be due to the differences in the TUN surgical technique (full- vs. split-thickness TUN preparation) and in the employed soft tissue graft (acellular dermal matrix vs. CTG). It may be speculated that the higher increase in KT observed in the present study in sites treated with TUN may be due to the secondary keratinization of the CTG left exposed. It is, however, undetermined whether this difference will be maintained over time, since there is evidence that KT may increase up to 9 years after surgery when CAF is combined with a CTG [42].

Although one out of three teeth treated with TUN showed uncovered CEJ during early healing (1–4 weeks), at 6 months around 80% of the sites demonstrated CRC, which indicates the occurrence of creeping attachment on teeth treated with this surgical technique. Conversely, teeth treated with CAF showed an opposite trend, since CRC decreased from 97.6% at week 1 to 78.1% at 6 months.

In the present study, patients operated with the test intervention (TUN) reported better PROMs and experienced a faster surgical intervention (19-min difference). These results are not consistent with a previous RCT reporting shorter surgery duration and lesser morbidity in patients treated with CAF [28]. These differences may be due to the different TUN technique employed in the referred study, involving the periosteal elevation and the coronal advancement of the gingival margins, which may have resulted in an additional surgical time and an increased postoperative morbidity [28].

In this RCT, we tried to standardize the CTG harvesting procedure between groups, by undertaking the randomization once the CTG was harvested. However, the CAF surgical technique has been recently modified by advocating a minimal-size application of CTG [43]. It is, therefore, yet to be determined whether this reduced-size CTG associated with CAF may compensate the differences in terms of surgical time and intra-/postoperative morbidity between groups observed in the present study.

The results of this trial answer to a clinically relevant question, and they are supported by a solid study design. However, some limitations should be taken into account, including the lack of blinding of the outcome assessors and the short follow-up (6 months). Moreover, despite a sample size calculation was performed, the statistical power may have been limited to detect smaller—yet clinically relevant—differences between groups on the primary outcome. Furthermore, data on type of GR (RT1/RT2) and gingival thickness was not collected, preventing the verification of a balanced distribution between groups of these prognostic parameters. Finally, both surgical operators were trained and experienced with the use of both techniques which—despite the dual-center setting—may reduce the whole generalizability of the present findings.

## Conclusions

The results of this dual-center RCT have shown that both surgical interventions (TUN and CAF) resulted in similar clinical outcomes for the treatment of multiple gingival recessions; however, TUN resulted in a significantly higher increase in keratinized tissue, in less patient morbidity, and in a shorter surgery duration.

## Supplementary Information

Below is the link to the electronic supplementary material.

Supplementary file1 (DOCX 234 KB)

## Data Availability

The datasets used and/or analyzed during the current study are available from the corresponding author upon reasonable request.
