# Supplementary material for: Tunnel vs. coronally advanced flap in combination with a connective tissue graft for the treatment of multiple gingival recessions: a multi-center randomized clinical trial
Source: Clin Oral Investig. 2023 Mar 29;27(7):3627–38. doi: 10.1007/s00784-023-04975-7 (PMC10329586; doi:10.1007/s00784-023-04975-7)
Supplement: Supplementary file 1 — Supplementary file1 (DOCX 234 KB) [file 784_2023_4975_MOESM1_ESM.docx]

**Table S1.** Intra-surgical patient-reported outcomes.

|  | **Overall**  (N=29) | **CAF**  (N=14) | **TUN**  (N=15) | **Effect size** | **p-value** |
| --- | --- | --- | --- | --- | --- |
| **Surgery perception** (VAS), mean (± SD) | 57.7 (±25.4) | 66.0 (±28.2) | 49.6 (±20.4) | MD= -16.0  (95% CI: -34.7/2.6) | 0.089 |
| **Intra-surgical pain** (VAS), mean (±SD) | 24.4 (±20.6) | 32.9 (±25.7) | 16.4 (±9.5) | MD= -16.4  (95% CI: -31.0/ -1.9) | ***0.028**** |

*Footnote:*
CAF, Coronally Advanced Flap; CI, confidence interval; TUN, Tunnel; MD, difference of means; SD, standard deviation; VAS, Visual Analog Scale.

* statistically significant

**Table S2.** Worst pain during the 14 postoperative days.

|  | **Overall**  (N=29) | **CAF**  (N=14) | **TUN**  (N=15) | **Effect size** | **p-value** |
| --- | --- | --- | --- | --- | --- |
| **Pain (worst) Day 1,** mean (±SD) | 53.7 (±19.3) | 61.0 (±20.6) | 46.8 (±15.7) | MD=-14.2  (95% CI: -28.1/-0.3) | ***0.045**** |
| **Pain (worst) Day 2,** mean (±SD) | 52.3 (±22.4) | 59.9 (±26.6) | 45.2 (±15.2) | MD=-14.8  (95% CI: -31.1/1.6) | 0.075 |
| **Pain (worst) Day 3,** mean (±SD) | 52.7 (±21.3) | 59.7 (±24.5) | 46.2 (±15.9) | MD=-13.4  (95% CI: -29.1/2.2) | 0.089 |
| **Pain (worst) Day 4,** mean (±SD) | 49.1 (±22.9) | 54.9 (±25.1) | 43.7 (±20.1) | MD=-11.1  (95% CI: -28.4/6.1) | 0.196 |
| **Pain (worst) Day 5,** mean (±SD) | 47.9 (±23.1) | 50.3 (±25.5) | 45.7 (±21.3) | MD=-4.6  (95% CI: -22.5/13.3) | 0.601 |
| **Pain (worst) Day 6,** mean (±SD) | 37.3 (±23.6) | 41.5 (±28.2) | 33.4 (±18.4) | MD=-8.1  (95% CI: -26.1/9.9) | 0.364 |
| **Pain (worst) Day 7,** mean (±SD) | 30.3 (±24.5) | 36.5 (±26.7) | 24.5 (±21.7) | MD=-11.9  (95% CI: -30.4/6.5) | 0.196 |
| **Pain (worst) Day 8,** mean (±SD) | 27.0 (±27.6) | 36.5 (±32.9) | 18.2 (±18.6) | MD=-18.3  (95% CI: -38.4/1.9) | 0.074 |
| **Pain (worst) Day 9,** mean (±SD) | 21.6 (±22.9) | 28.6 (±28.6) | 15.2 (±13.9) | MD= -13.4  (95% CI: -30.4/3.5) | 0.116 |
| **Pain (worst) Day 10,** mean (±SD) | 20.9 (±21.8) | 29.6 (±28.4) | 12.9 (±7.3) | MD=-16.7  (95% CI: -32.3/-1.2) | ***0.036**** |
| **Pain (worst) Day 11,** mean (±SD) | 17.5 (±17.6) | 24.1 (±23.3) | 11.3 (±5.6) | MD=-12.8  (95% CI: -25.5/-0.1) | ***0.048**** |
| **Pain (worst) Day 12,** mean (±SD) | 11.6 (±15.4) | 16.5 (±20.3) | 6.9 (±6.4) | MD=-9.6  (95% CI: -20.9/1.7) | 0.094 |
| **Pain (worst) Day 13,** mean (±SD) | 8.4 (14.8) | 13.0 (±19.4) | 4.1 (±6.9) | MD=-8.9  (95% CI: -19.8/2.1) | 0.108 |
| **Pain (worst) Day 14,** mean (±SD) | 6.4 (±14.6) | 10.7 (±19.9) | 2.4 (±4.6) | MD=-8.3  (95% CI: -19.1/2.6) | 0.130 |
| **Time to no significant pain (worst) (VAS<10)**** (days), mean (±SD) | 12.2 (±3.1) | 12.8 (±2.6) | 11.6 (±3.5) | MD=-1.2  (95% CI: -3.5/1.2) | 0.312 |
| **Time to no pain (worst) (VAS=0)** * * (days), mean (±SD) | 13.1 (±2.2) | 13.1 (±2.6) | 13.1 (±1.8) | MD=0.06  (95% CI: -1.6/1.8) | 0.940 |

*Footnote:*
CAF, Coronally Advanced Flap; CI, confidence interval; MD, difference in means; N, number; SD, Standard Deviation; TUN, Tunnel.

* statistically significant

** if the value was not reached at day 14, it was considered as “15”

**Table S3.** Average pain during the 14 postoperative days.

|  | **Overall**  (N=29) | **CAF**  (N=14) | **TUN**  (N=15) | **Effect size** | **p-value** |
| --- | --- | --- | --- | --- | --- |
| **Pain (average) Day 1,** mean (±SD) | 49.3 (±19.3) | 55.7 (±20.6) | 43.4 (±16.4) | MD=-12.3  (95% CI: -26.5/1.8) | 0.085 |
| **Pain (average) Day 2,** mean (±SD) | 46.8 (±20.5) | 52.8 (±23.2) | 41.3 (±16.5) | MD=-11.6  (95% CI: -26.8/3.7) | 0.132 |
| **Pain (average) Day 3,** mean (±SD) | 46.7 (±20.6) | 51.7 (±23.8) | 42.1 (±16.5) | MD=-9.7  (95% CI: -25.2/5.9) | 0.212 |
| **Pain (average) Day 4,** mean (±SD) | 43.3 (±20.8) | 48.4 (±22.7) | 38.5 (±18.3) | MD=-9.8  (95% CI: -25.5/5.8) | 0.210 |
| **Pain (average) Day 5,** mean (±SD) | 44.8 (±23.6) | 46.8 (±26.7) | 42.9 (±21.0) | MD=-3.9  (95% CI: -22.2/14.3) | 0.663 |
| **Pain (average) Day 6,** mean (±SD) | 37.2 (±25.6) | 44.2 (±29.2) | 30.7 (±20.6) | MD=-13.5  (95% CI: -32.7/5.6) | 0.158 |
| **Pain (average) Day 7,** mean (±SD) | 26.9 (±24.5) | 32.6 (±27.3) | 21.8 (±21.2) | MD=-10.8  (95% CI: -29.3/7.8) | 0.244 |
| **Pain (average) Day 8,** mean (±SD) | 23.8 (±26.7) | 31.4 (±32.1) | 16.7 (±18.9) | MD=-14.7  (95% CI: -34.7/5.2) | 0.141 |
| **Pain (average) Day 9,** mean (±SD) | 19.3 (±21.7) | 24.3 (27.4) | 14.7 (±14.0) | MD=-9.7  (95% CI: -26.1/6.8) | 0.238 |
| **Pain (average) Day 10,** mean (±SD) | 17.7 (±17.9) | 23.8 (±23.6) | 11.9 (±7.4) | MD=-11.8  (95% CI: -24.9/1.3) | 0.076 |
| **Pain (average) Day 11,** mean (±SD) | 15.8 (±16.4) | 20.8 (±22.2) | 11.0 (±5.6) | MD=-9.8  (95% CI: -21.9/2.3) | 0.109 |
| **Pain (average) Day 12,** mean (±SD) | 9.6 (±12.5) | 13.0 (±16.4) | 6.4 (±6.3) | MD=-6.6  (95% CI: -15.9/2.7) | 0.157 |
| **Pain (average) Day 13,** mean (±SD) | 7.4 (±12.6) | 10.9 (±16.2) | 4.1 (±6.9) | MD=-6.7  (95% CI: -16.1/2.6) | 0.152 |
| **Pain (average) Day 14,** mean (±SD) | 5.6 (±11.6) | 8.9 (±15.5) | 2.4 (±4.6) | MD=-6.6  (95% CI: -15.1/2.0) | 0.129 |

*Footnote:*
CAF, Coronally Advanced Flap; CI, confidence interval; MD, difference in means; N, number; SD, Standard Deviation; TUN, Tunnel.

**Table S4.** Current pain during the 14 postoperative days.

|  | **Overall**  (N=29) | **CAF**  (N=14) | **TUN**  (N=15) | **Effect size** | **p-value** |
| --- | --- | --- | --- | --- | --- |
| **Pain (current) Day 1,** mean (±SD) | 44.7 (±22.9) | 49.5 (±27.5) | 40.3 (±17.4) | MD=-9.2  (95% CI: -26.6/8.2) | 0.289 |
| **Pain (current) Day 2,** mean (±SD) | 43.0 (±21.3) | 45.7 (±26.3) | 40.5 (±15.8) | MD=-5.2  (95% CI: -21.6/11.2) | 0.518 |
| **Pain (current) Day 3,** mean (±SD) | 41.7 (±20.8) | 45.3 (±23.3) | 38.4 (±18.2) | MD=-7.0  (95% CI: -22.9/8.9) | 0.374 |
| **Pain (current) Day 4,** mean (±SD) | 42.9 (±21.2) | 48.6 (±24.4) | 37.7 (±16.8) | MD=-10.9  (95% CI: -26.7/4.9) | 0.171 |
| **Pain (current) Day 5,** mean (±SD) | 42.1 (±24.8) | 43.5 (±28.3) | 40.9 (±22.1) | MD=-2.6  (95% CI: -21.9/16.7) | 0.784 |
| **Pain (current) Day 6,** mean (±SD) | 34.6 (±22.4) | 40.1 (±25.3) | 29.5 (±18.6) | MD=-10.6  (95% CI: -27.4/6.2) | 0.207 |
| **Pain (current) Day 7,** mean (±SD) | 26.8 (±24.9) | 32.0 (±27.8) | 21.9 (±21.7) | MD=-10.1  (95% CI: -29.0/8.8) | 0.284 |
| **Pain (current) Day 8,** mean (±SD) | 24.1 (±27.5) | 32.3 (±32.9) | 16.5 (±19.3) | MD=-15.7  (95% CI: -36.2/4.7) | 0.125 |
| **Pain (current) Day 9,** mean (±SD) | 19.5 (±21.7) | 24.8 (±27.3) | 14.5 (±14.1) | MD=-10.3  (95% CI: -26.7/6.0) | 0.206 |
| **Pain (current) Day 10,** mean (±SD) | 11.6 (±22.1) | 18.1 (±29.3) | 5.6 (±10.2) | MD=-12.4  (95% CI: -28.9/4.1) | 0.134 |
| **Pain (current) Day 11,** mean (±SD) | 9.8 (±17.8) | 16.1 (±23.1) | 3.9 (±7.9) | MD=-12.2  (95% CI: -25.2/0.8) | 0.065 |
| **Pain (current) Day 12,** mean (±SD) | 7.6 (±14.5) | 12.4 (±18.8) | 3.1 (±6.8) | MD=-9.2  (95% CI: -19.8/1.4) | 0.086 |
| **Pain (current) Day 13,** mean (±SD) | 6.3 (±11.8) | 9.7 (±14.9) | 3.1 (±6.8) | MD=-6.7  (95% CI: -15.4/2.1) | 0.130 |
| **Pain (current) Day 14,** mean (±SD) | 3.9 (±8.4) | 6.1 (±11.0) | 2.0 (±4.6) | MD=-4.1  (95% CI: -10.5/2.2) | 0.195 |

*Footnote:*
CAF, Coronally Advanced Flap; CI, confidence interval; MD, difference in means; N, number; SD, Standard Deviation; TUN, Tunnel.

**Table S5.** Medications consumption (pain-killers and/or anti-inflammatory drugs) during the 14 postoperative days.

|  | **Overall**  (N=29) | **CAF**  (N=14) | **TUN**  (N=15) | **Effect size** | **p-value** |
| --- | --- | --- | --- | --- | --- |
| **At least 1 medication - Day 1,** N (%) | 29 (100) | 14 (100) | 15 (100) | NE | - |
| **At least 1 medication - Day 2,** N (%) | 27 (100) | 14 (100) | 13 (100) | NE | - |
| **At least 1 medication - Day 3,** N (%) | 27 (93.1) | 14 (100) | 13 (96.7) | NE | - |
| **At least 1 medication - Day 4,** N (%) | 26 (92.9) | 14 (100) | 12 (85.7) | NE | - |
| **At least 1 medication - Day 5,** N (%) | 26 (89.7) | 14 (100) | 12 (80.0) | NE | - |
| **At least 1 medication - Day 6,** N (%) | 24 (82.8) | 12 (85.7) | 12 (80.0) | OR=0.5  (95% CI: 0.04/6.2) | 0.590 |
| **At least 1 medication - Day 7,** N (%) | 14 (48.3) | 10 (71.4) | 4 (26.7) | OR=0.2  (95% CI: 0.04/0.9) | ***0.046**** |
| **At least 1 medication - Day 8,** N (%) | 10 (34.5) | 9 (64.3) | 1 (6.7) | OR=0.1  (95% CI: 0.01/0.5) | ***0.009**** |
| **At least 1 medication - Day 9,** N (%) | 7 (24.1) | 6 (42.9) | 1 (6.7) | OR=0.2  (95% CI: 0.03/1.3) | 0.089 |
| **At least 1 medication - Day 10,** N (%) | 7 (24.1) | 6 (42.9) | 1 (6.7) | OR=0.2  (95% CI: 0.03/1.3) | 0.089 |
| **At least 1 medication - Day 11,** N (%) | 5 (17.2) | 5 (35.7) | 0 (0.0) | OR=0.1  (95% CI: 0.02/1.3) | 0.079 |
| **At least 1 medication - Day 12,** N (%) | 5 (17.2) | 5 (35.7) | 0 (0.0) | OR=0.1  (95% CI: 0.01/1.3) | 0.081 |
| **At least 1 medication - Day 13,** N (%) | 5 (17.2) | 5 (35.7) | 0 (0.0) | NE | - |
| **At least 1 medication - Day 14,** N (%) | 3 (10.3) | 3 (21.4) | 0 (0.0) | NE | - |
| **Days on medications (days)**, mean (±SD) | 7.6 (±3.1) | 9.1 (±3.3) | 6.0 (±2.0) | MD=-3.1  (95% CI: -5.4/-0.8) | ***0.011**** |

*Footnote:*
CAF, Coronally Advanced Flap; CI, confidence interval; MD, difference in means; N, number; NE, not estimable; OR, odds ratio; SD, Standard Deviation; TUN, Tunnel; %, percentage.

* statistically significant

**Table S6.** PREMs during the 14 postoperative days.

|  | **Overall**  (N=29) | **CAF**  (N=14) | **TUN**  (N=15) | **Effect size** | **p-value** |
| --- | --- | --- | --- | --- | --- |
| **PREMs Day 1,** mean (±SD) | 36.9 (±11.3) | 39.6 (±12.3) | 34.3 (±10.1) | MD= -5.2  (95% CI: -13.8/3.3) | 0.219 |
| **PREMs Day 2,** mean (±SD) | 36.9 (±12.2) | 40.9 (±12.1) | 33.3 (±11.4) | MD= -7.6  (95% CI: -16.5/1.4) | 0.093 |
| **PREMs Day 3,** mean (±SD) | 36.1 (±13.0) | 40.1 (±13.2) | 32.2 (±12.1) | MD= -7.7  (95% CI: -17.4/1.9) | 0.112 |
| **PREMs Day 4,** mean (±SD) | 32.9 (±14.9) | 35.1 (±16.0) | 31.0 (±14.0) | MD= -4.1  (95% CI: -15.5/7.4) | 0.472 |
| **PREMs Day 5,** mean (±SD) | 30.9 (±15.5) | 31.8 (±18.8) | 30.0 (±12.3) | MD =-1.8  (95% CI: -13.8/10.2) | 0.762 |
| **PREMs Day 6,** mean (±SD) | 24.4 (±14.5) | 28.9 (±17.3) | 20.2 (±10.1) | MD= -8.7  (95% CI: -19.4/1.9) | 0.106 |
| **PREMs Day 7,** mean (±SD) | 21.7 (±14.0) | 26.8 (±16.7) | 16.9 (±9.1) | MD= -9.9  (95% CI: -20.1/0.2) | 0.055 |
| **PREMs Day 8,** mean (±SD) | 14.4 (±15.6) | 19.4 (±18.0) | 9.7 (±11.6) | MD= -9.8  (95% CI: -21.2/1.7) | 0.092 |
| **PREMs Day 9,** mean (±SD) | 13.4 (±14.9) | 18.9 (±18.4) | 8.3 (±8.3) | MD= -10.6  (95 % CI: -21.4/0.2) | 0.053 |
| **PREMs Day 10,** mean (±SD) | 8.1 (±15.4) | 13.9 (±20.2) | 2.7 (±5.5) | MD= -11.2  (95% CI: -22.3/-0.1) | ***0.048**** |
| **PREMs Day 11,** mean (±SD) | 6.2 (±12.7) | 10.6 (±16.9) | 2.1 (±4.6) | MD= -8.6  (95% CI: -17.8/0.7) | 0.068 |
| **PREMs Day 12,** mean (±SD) | 6.2 (±13.5) | 11.0 (±17.6) | 1.7 (±5.4) | MD= -9.3  (95% CI: -19.1/0.5) | 0.061 |
| **PREMs Day 13,** mean (±SD) | 5.8 (±12.9) | 10.1 (±17.0) | 1.7 (±5.4) | MD= -8.5  (95% CI: -17.9/1.0) | 0.078 |
| **PREMs Day 14,** mean (±SD) | 5.1 (±11.9) | 9.7 (±16.1) | 0.9 (±2.4) | MD= -8.8  (95% CI: -17.4/-0.3) | ***0.044**** |

*Footnote:*
CAF, Coronally Advanced Flap; CI, confidence interval; MD, difference in means; N, number; SD, Standard Deviation; TUN, Tunnel.

*** statistically significant

**Table S7.** OHIP-14 during the 14 postoperative days.

|  | **Overall**  (N=29) | **CAF**  (N=14) | **TUN**  (N=15) | **Effect size** | **p-value** |
| --- | --- | --- | --- | --- | --- |
| **OHIP-14 Day 1,** mean (±SD) | 22.2 (±11.1) | 23.9 (±13.6) | 20.7 (±8.3) | MD= -3.1  (95% CI: -11.6/5.4) | 0.458 |
| **OHIP-14 Day 2,** mean (±SD) | 21.1 (±11.4) | 21.8 (±13.4) | 20.4 (±9.5) | MD= -1.4  (95% CI: -10.2/7.4) | 0.749 |
| **OHIP-14 Day 3,** mean (±SD) | 20.8 (±11.1) | 22.1 (±13.0) | 19.5 (±9.2) | MD= -2.5  (95% CI: -11.1/6.0) | 0.547 |
| **OHIP-14 Day 4,** mean (±SD) | 19.8 (±11.1) | 21.1 (±12.3) | 18.7 (±10.1) | MD= -2.4  (95% CI: -10.9/6.1) | 0.568 |
| **OHIP-14 Day 5,** mean (±SD) | 20.7 (±13.6) | 20.9 (±12.8) | 20.5 (±14.8) | MD= -0.3  (95% CI: -10.9/10.2) | 0.950 |
| **OHIP-14 Day 6,** mean (±SD) | 16.4 (±11.3) | 17.8 (±13.5) | 15.1 (±8.9) | MD= -2.7  (95% CI: -11.4/5.9) | 0.525 |
| **OHIP-14 Day 7,** mean (±SD) | 15.8 (±12.0) | 17.2 (±15.3) | 14.5 (±8.2) | MD= -2.7  (95% CI: -11.9/6.6) | 0.558 |
| **OHIP-14 Day 8,** mean (±SD) | 11.8 (±14.1) | 15.0 (±17.3) | 8.8 (±10.1) | MD= -6.2  (95% CI: -16.9/4.5) | 0.245 |
| **OHIP-14 Day 9,** mean (±SD) | 10.6 (±11.9) | 13.3 (±14.7) | 8.0 (±8.4) | MD= -5.3  (95% CI: -14.3/3.8) | 0.241 |
| **OHIP-14 Day 10,** mean (±SD) | 7.9 (±14.4) | 13.3 (±14.9) | 3.0 (±7.8) | MD= -10.3  (95% CI: -20.7/0.11) | 0.052 |
| **OHIP-14 Day 11,** mean (±SD) | 7.4 (±15.8) | 13.8 (±20.7) | 1.5 (±5.2) | MD= -12.3  (95% CI: -23.5/-0.9) | ***0.035**** |
| **OHIP-14 Day 12,** mean (±SD) | 4.9 (11.5) | 9.1 (±15.3) | 0.9 (±3.3) | MD= -8.2  (95% CI: -16.5/0.1) | 0.053 |
| **OHIP-14 Day 13,** mean (±SD) | 5.2 (±12.0) | 9.8 (±15.9) | 0.9 (±3.4) | MD= -8.9  (95% CI: -17.5/-0.3) | ***0.043**** |
| **OHIP-14 Day 14,** mean (±SD) | 4.0 (±10.4) | 8.3 (±14.0) | 0.0 (±0.0) | MD= -8.3  (95% CI: -15.7/-0.9) | ***0.030**** |

*Footnote:*
CAF, Coronally Advanced Flap; MD, difference in means; N, number; OHIP, Oral Health Impact Profile; SD, Standard Deviation; TUN, Tunnel.

**Table S8.** OHIP-14 at 6 months after surgery.

|  | **Overall**  (N=29) | **CAF**  (N=14) | **TUN**  (N=15) | **Effect size** | **p-value** |
| --- | --- | --- | --- | --- | --- |
| **OHIP-14,** mean (±SD) |  |  |  |  |  |
| Functional limitation | 0.2 (±0.5) | 0.2 (±0.6) | 0.1 (±0.5) | MD= -0.08  (95% CI: -0.5/0.3) | 0.694 |
| Physical pain | 0.5 (±1.1) | 0.7 (±1.3) | 0.3 (±0.9) | MD= -0.4  (95% CI: -1.2/0.5) | 0.370 |
| Psychological discomfort | 2.1 (±1.4) | 2.1 (±1.4) | 2.1 (±1.4) | MD= 0.06  (95% CI: -1.0/1.1) | 0.906 |
| Physical disability | 0.4 (±0.9) | 0.4 (±0.9) | 0.4 (0.8) | MD= -0.03  (95% CI: -0.7/0.6) | 0.931 |
| Psychological disability | 0.3 (±0.8) | 0.3 (±0.6) | 0.3 (±1.0) | MD= -0.02  (95% CI: -0.7/0.6) | 0.953 |
| Social disability | 0.1 (±0.5) | 0.3 (±0.7) | 0.0 (±0.0) | MD= -0.3  (95% CI: -0.7/0.1) | 0.139 |
| Handicap | 0.1 (±0.4) | 0.2 (±0.6) | 0.0 (±0.0) | MD= -0.2  (95% CI: -0.5/0.1) | 0.163 |
| **OHIP-14 Total**, mean (±SD) | 3.7 (±3.7) | 4.2 (±4.3) | 3.6 (±3.3) | MD= -0.9  (95% CI: -3.8/1.9) | 0.504 |

*Footnote:*
CAF, Coronally Advanced Flap; CI, confidence interval; MD, difference in means; N, number; OHIP-14, Oral-Health Impact Profile-14; SD, Standard Deviation; TUN, Tunnel.

**Table S9.** Condition-specific HRQoL at 6 months after surgery.

|  | **Overall**  (N=29) | **CAF**  (N=14) | **TUN**  (N=15) | **Effect size** | **p-value** |
| --- | --- | --- | --- | --- | --- |
| **Condition-specific HRQoL,** mean (±SD) |  |  |  |  |  |
| Esthetics | 2.1 (±1.1) | 2.2 (±1.2) | 2 (±1.1) | MD= -0.2  (95% CI: -1.1/0.7) | 0.623 |
| Cold sensitivity | 2.2 (±1.3) | 1.9 (±1.5) | 2.4 (±1.2) | MD= 0.5  (95% CI: -0.6/1.5) | 0.352 |
| Brushing sensitivity | 2.1 (±1.3) | 1.9 (±1.5) | 2.3 (±1.1) | MD= 0.5  (95% CI: -0.5/1.5) | 0.330 |
| Tooth wear | 1.9 (±1.0) | 1.9 (1.2) | 1.9 (±0.9) | MD= 0.08  (95% CI: -0.7/0.9) | 0.849 |
| Dental caries | 2.3 (±1.3) | 2.2 (1.4) | 2.4 (±1.2) | MD= 0.2  (95% CI: -0.8/1.20) | 0.711 |
| Fear of losing teeth | 1.3 (±1.5) | 1.6 (1.7) | 1.1 (±1.2) | MD= -0.6  (95% CI: -1.7/0.5) | 0.300 |
| **Condition-specific HRQoL Total,** mean (±SD) | 11.9 (±6.3) | 11.7 (±7.0) | 12.1 (±5.8) | MD= 0.4  (95% CI: -4.5/5.3) | 0.862 |

*Footnote:*
CAF, Coronally Advanced Flap; CI, confidence interval; HRQoL, Health-Related Quality of Life; MD, difference in means; N, number; SD, Standard Deviation; TUN, Tunnel.

**Figure S1.** CONSORT 2010 Flow Diagram.
